# Effectiveness of Carbon Ion Radiation in Locally Advanced Pancreatic Cancer

**DOI:** 10.3389/fonc.2021.708884

**Published:** 2021-07-14

**Authors:** Jakob Liermann, Patrick Naumann, Fabian Weykamp, Philipp Hoegen, Juergen Debus, Klaus Herfarth

**Affiliations:** ^1^ Department of Radiation Oncology, Heidelberg University Hospital, Heidelberg, Germany; ^2^ Heidelberg Institute for Radiation Oncology (HIRO), Heidelberg, Germany; ^3^ National Center for Tumor Diseases (NCT), Heidelberg, Germany; ^4^ Clinical Cooperation Unit Radiation Oncology, German Cancer Research Center (DKFZ), Heidelberg, Germany; ^5^ Heidelberg Ion Beam Therapy Center, Heidelberg, Germany; ^6^ German Cancer Consortium (DKTK), partner site Heidelberg, German Cancer Research Center (DKFZ), Heidelberg, Germany

**Keywords:** pancreatic cancer, carbon ion radiotherapy, particle therapy, locally advanced pancreatic cancer, irradiation, heavy ion

## Abstract

**Purpose:**

Effective treatment strategies for unresectable locally advanced pancreatic cancer (LAPC) patients are eagerly warranted. Recently, convincing oncological outcomes were demonstrated by carbon ion radiotherapy. Nevertheless, there is a lack of evidence for this modern radiation technique due to the limited number of carbon ion facilities worldwide. Here, we analyze feasibility and efficacy of carbon ion radiotherapy in the management of LAPC at Heidelberg Ion Beam Therapy Center (HIT).

**Methods:**

Between 2015 and 2020, 21 LAPC patients were irradiated with carbon ions with a total dose of 48 Gy (RBE) in single doses of 4 Gy (RBE). Three patients (14%) were treated with concomitant chemotherapy with gemcitabine 300 mg/m^2^ body surface weekly. Toxicity rates were extracted from the charts. Overall survival, progression free survival, local control, and locoregional control were evaluated using Kaplan–Meier estimates.

**Results:**

One patient developed ascites CTCAE grade III during radiotherapy, which was related to a later histologically confirmed metachronous peritoneal carcinomatosis. No further higher-graded toxicity could be observed. The most common symptoms were nausea and abdominal pain. After a median estimated follow-up time of 19.1 months, the median progression free survival was 3.7 months, and the median overall survival was 11.9 months. The estimated 1-year local control and locoregional control rates were 89 and 84%, respectively.

**Conclusion:**

Carbon ion radiotherapy of LAPC patients is safely feasible. Local tumor control rates were high. Nevertheless, compared to historical data, an overall survival improvement could not be observed. This could be explained by the poor prognosis of the selected underlying patients that mostly did not respond to prior chemotherapy as well as the early and frequent emergence of distant metastases that demonstrate the necessity of additional chemotherapy in further studies.

## Introduction

In pancreatic cancer, there is a lack of effective therapy options. Over the last decades, the limited five-year overall survival rate of 5–10% (1) could only be marginally challenged by modern treatment strategies. So far, surgical resection is the only curative therapy (2). However, in the majority of the patients, the tumor is deemed unresectable due to distant metastases or due to vessel involvement. Recently, Iacobuzio-Donahue et al. demonstrated local disease burden to be the cause of approximately one third of all pancreatic cancer related deaths (3). These findings demonstrate the urgent need for effective local treatment strategies.

In the last decades, several approaches tried to improve the oncological outcome of patients suffering from locally advanced pancreatic cancer (LAPC). One important goal in LAPC therapy is downstaging to gain secondary resectability. Because of the promising local effects of radiotherapy, multiple trials tested a potential benefit of chemoradiation over chemotherapy alone (4–6), but the data remain inconclusive. The Eastern Cooperative Oncology Group (ECOG) trial E4201 (6) and the Gastrointestinal Tumor Study Group (GITISG) trial 9283 (7) showed a slight beneficial effect of chemoradiation over chemotherapy. However, the LAP07 trial (4) revealed no difference in the median overall survival, although local tumor progression was significantly lower after chemoradiation compared to chemotherapy alone (32% *vs*. 46%). The efficacy of chemotherapy could also be improved, recently. The most effective chemotherapy regime consisting of folinic acid, fluorouracil, irinotecan, and oxaliplatin (FOLFIRINOX) as part of LAPC therapy is correlated with a median overall survival of 24.2 months (8). However, many patients do not tolerate this aggressive chemotherapy regime due to comorbidity. As the results of chemoradiotherapy remain controversial, there is an ongoing discussion on the role of chemoradiation in the management of LAPC.

The observed limited effect of conventional radiotherapy in pancreatic cancer could partially be explained by the relatively low radiation doses applicable. This is due to the gastrointestinal tract which is highly sensitive to radiotherapy and which is situated adjacent to the pancreas (9, 10). To improve radiotherapy in LAPC, radiation doses should be increased. This could be reached by modern radiation techniques, such as stereotactic body radiotherapy, magnetic resonance (MR)-guided radiotherapy or particle therapy (11–14). Using these techniques, higher doses can be applied to the tumor while respecting the dose limits for the adjacent organs at risk (OARs). Recently, Shinoto et al. presented convincing results of carbon ion radiotherapy in LAPC (15). In a dose-escalating trial, the observed median overall survival was 19.6 months after chemoradiation with 43.2–55.2 Gy (RBE) carbon ions applied in 12 fractions and combined with gemcitabine.

Carbon ion radiotherapy is known for two major advantages over conventional photon radiotherapy. First, there are physical characteristics making carbon ion radiotherapy highly conformal and precise. Energy deposition of particle therapy in irradiated tissue is different to the one of photon radiotherapy. Within a particle beam, there is a relatively low energy deposition in the entrance channel. The particles slow down and finally stop in a calculable depth, depending on their speed, represented by an increase of energy deposition and resulting in a maximum of energy deposition at a certain depth, the so-called Bragg Peak (16). There is almost no energy deposition behind this Bragg Peak. The resulting dose gradients in particle therapy are therefore very steep which makes the dose application highly precise. Second, carbon ions are known for a higher linear energy transfer (LET) and a higher relative biological effectiveness (RBE) compared to photons and even compared to protons (17). This is a biological advantage over photon radiotherapy as carbon ions are *e.g.* not as oxygen-dependent as photons. The biological characteristics of carbon ions could translate in improved therapy outcomes in so-far deemed radioresistant tumors (18).

In carbon ion radiotherapy planning, one of the most crucial aspects is the multifactorial dependency of the RBE resulting in different RBE values within the beam (19). Different carbon ion facilities use different RBE-models for carbon ion radiotherapy planning. Therefore, dose and therapy concepts cannot simply be transferred from one facility to another (20).

The advantages of carbon ion radiotherapy over conventional photon radiotherapy could improve oncological outcomes of LAPC patients as demonstrated by Shinoto et al. (15) In the present study, we analyze the feasibility and the efficacy of this promising radiation technique in the treatment of LAPC patients at Heidelberg Ion Beam Therapy Center (HIT).

## Methods

### Patients

All patients presented with histologically confirmed pancreatic ductal adenocarcinoma or suspicious pancreatic tumor in imaging with correlating elevated tumor markers. To be included in the study, patients needed to suffer from inoperable LAPC without any sign of distant metastases (American Joint Committee on Cancer stage III). Two exceptions were made. One patient presented with a radiological suspicion of a distant lymph node metastasis, but the metastasis was not histologically confirmed at the time of radiotherapy. Another patient was included in the study, although he presented with hepatic metastases due to the fact that the hepatic metastases responded excellently to initial chemotherapy. A certain distance between the gastrointestinal tract and the tumor was not required. Any type and duration of prior chemotherapy was allowed. Recurrent cancer cases and patients participating in the ongoing PACK-trial (21) were excluded from the analysis. The inclusion criteria were chosen widely, as the institutional LAPC patient cohort treated with carbon ion radiotherapy is too small to define more specific subgroups.

### Target Volume Definition

Planning imaging for radiotherapy was performed using four-dimensional native CT scans with a slice thickness of 3 mm to consider respiratory movement. Contrast-enhanced images were generated for differentiation of tumor and healthy tissue in delineation. Additionally, in six cases (29%), fibroblast activation protein inhibitor-positron emission tomography (FAPI-PET) was performed prior to radiotherapy to improve target definition as recently demonstrated for locally recurrent pancreatic cancer (22). Contouring and radiotherapy planning were performed with the treatment planning system Syngo PT Planning (Siemens, Erlangen, Germany).

For delineation of the gross tumor volume (GTV), all applicable imaging was used to define the macroscopic tumor. Assumed microscopic tumor invasion was defined as clinical target volume (CTV). Therefore, the GTV was isometrically expanded by 6 mm and corrected considering anatomic boundaries such as non-infiltrated bone. Locoregional lymph nodes and the neuro-plexus were only part of the CTV when infiltrated. Considering respiratory movement, an internal target volume (ITV) was generated. The planning target volume (PTV) consisted of the ITV, enlarged by 5 mm in all directions (7 mm in beam direction).

### Dose Prescription and Dose Constraints

**Figure 1:**
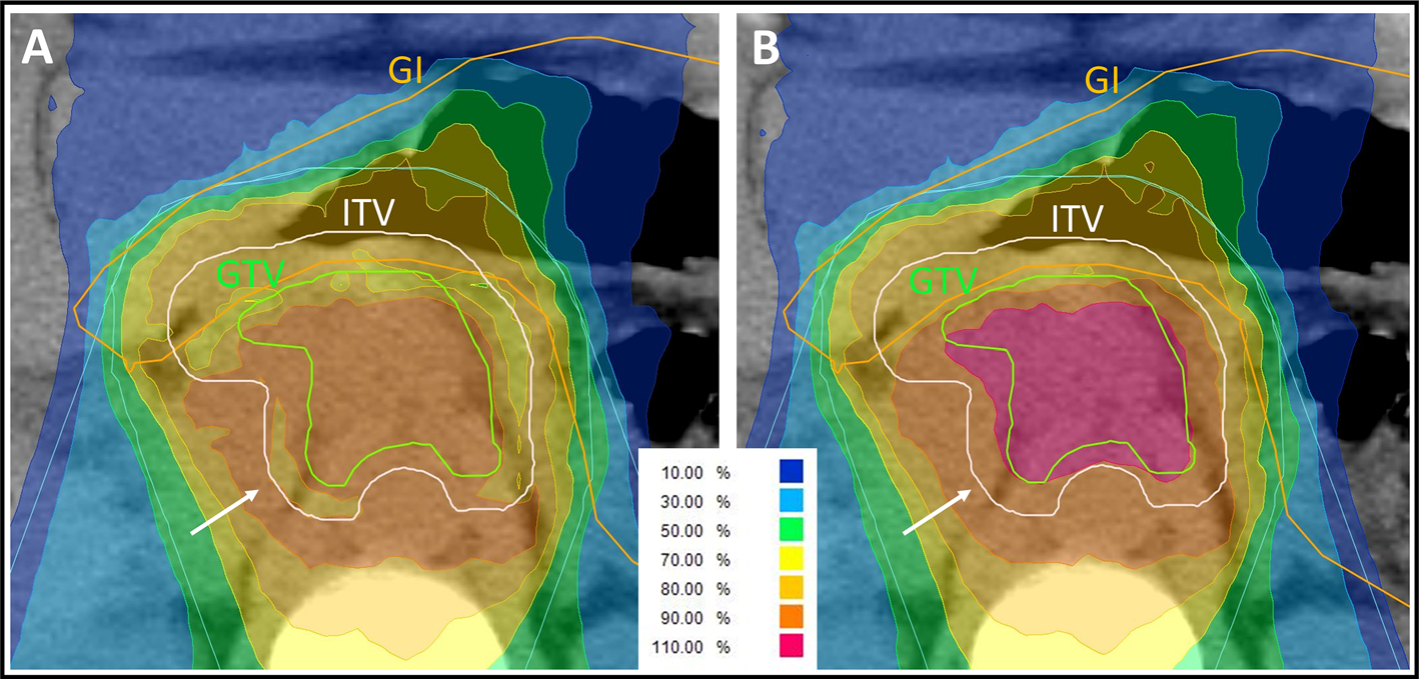
Representative carbon ion radiation plan of a locally advanced pancreatic cancer patient using a clinical *α*/*β*-ratio of 5 Gy for the internal target volume (ITV) and 2 Gy for the surrounding tissue in the treatment planning software’s integrated relative biological effectiveness (RBE) model. The isodose lines are demonstrated in different colors. The percentages of the isodose lines shown in the legend correspond to the prescribed dose of 48 Gy (RBE) in 12 fractions. Underdosage of the ITV and of the gross tumor volume (GTV) needed to be accepted to respect the gastrointestinal (GI) constraints. **(A)** Due to the *α*/*β*-ratio shift at the edge of the ITV, the peripheral ITV is irradiated with a lower biological dose than the surrounding tissue (white arrow), whereas the physically applied dose is increasing towards the center of the ITV. **(B)** Forward calculation using an *α*/*β*-ratio of 2 Gy for all volumes in the RBE model. The dose distribution at the edge of the ITV is more homogenous (white arrow) but in this plan presentation, the GTV seems to be overdosed. This forward calculation helps in analyzing the plan but is not assumed to be correct, because of the missing biological assumption of the higher *α*/*β*-ratio for the ITV.

Patients were irradiated with a total dose of 48 Gy (RBE) applied in 12 fractions. The corresponding equivalent dose at 2 Gy (EQD2) is 61.7 Gy, assuming an *α*/*β*-ratio of 5 Gy. A dose maximum in the upper gastrointestinal tract of 43.2 Gy (RBE) should be respected. Underdosage of the planning target volume (PTV) to respect gastrointestinal constraints in challenging cases was decided upon individually case by case. A representative underdosage within the target volume is demonstrated in **Figure 1**. The dosage of the spinal cord was restricted to a maximum of 36 Gy (RBE), and the kidney volume receiving more than 24 Gy (RBE) was not allowed to exceed 20% of the whole organ.

### Treatment Delivery

Carbon ion radiotherapy was applied with an intensity-controlled raster scanning system for beam application at a rotating gantry. The first six patients (29%) were situated in prone position to avoid a beam entry through the couch. After commissioning irradiation through the treatment table, the remaining 15 patients (71%) could be treated in supine position. Accordingly, patients were predominantly situated on their back with the arms over the head (in Wing-Step mount) and with support in the back of the knee (knee cushion or Pro-Step mount). The laser-marked reference spots were defined by three small ink marks (Beekley spots). In all but one cases, two dorsal oblique radiation beams were used. Four-dimensional CT data of each patient were analyzed to evaluate the respiratory movement. In cases with large cranio-caudal target movement, gating was considered. In cases with an acceptable movement of the target, an ITV of all respiration phases was generated instead. In the presented patient cohort, finally, none of the patients was irradiated in a gating technique. Abdominal compression was not used to avoid a forced proximity of the gastrointestinal to the target volume. Image-guidance was performed through daily orthogonal X-rays and regular CT scans (at least once per week). If available, operation clips or stents were used for alignment. Otherwise, the spine was used to match X-rays and planning CT images. To consider organ movement and anatomical changes, a planning risk volume of the gastrointestinal was generated based on the four-dimensional CT data and patients needed to fast for at least 3 h prior to irradiation. If necessary, a new plan was generated based on the performed control CT. In the presented analysis, this was the case in two patients.

### Treatment Planning

For biological plan optimization in carbon ion radiotherapy, different RBE values within the beam need to be considered. Therefore, the local effect model (LEM) I is integrated in the used treatment planning system (TPS). In LEM I, different *α*/*β*-ratios for the ITV (first three patients: *α*/*β* = 2 Gy, remaining 18 patients: *α*/*β* = 5 Gy) and for the surrounding tissues (*α*/*β* = 2 Gy) were used.

The implementation of two different *α*/*β*-ratios in the RBE model results in a complex dose distribution. In the ITV (*α*/*β* = 5 Gy), the physically applied dose needs to be higher than in the surrounding tissue (*α*/*β* = 2 Gy) to achieve the same biological dose. Thus, in the biological dose distribution prediction, there is a shift from the *α*/*β*-ratio of 2 Gy (within the surrounding tissue) to the *α*/*β*-ratio of 5 Gy (within the ITV). The biological dose in the peripheral margin of the ITV is deemed lower than the one of the surrounding tissue adjacent to the ITV edge (**Figure 1A**). This uncommon presentation of dose distribution seems inappropriate from the point of view of a conventional radiation oncologist. To facilitate plan evaluation, a forward calculation is performed using an *α*/*β*-ratio of 2 Gy in LEM I for all structures including the ITV and the surrounding tissue (**Figure 1B**). As a consequence, the biological dose within the ITV is much higher in this forward calculation and should not be taken for granted. On the other hand, dose distribution seems more appropriate at the edge of the ITV. For adequate plan evaluation, both the actual plan and the forward calculation should be analyzed.

### Follow-up and Response Evaluation

Follow-up was defined from the start of radiotherapy and was calculated using the reverse Kaplan–Meier method (23). Three-monthly contrast-enhanced CT scans and clinical visits were evaluated, whenever available. RECIST 1.1.-criteria were used in CT-based response evaluation. In-field tumor progression was denominated as “local tumor recurrence”. “Regional tumor recurrence” was defined as out-field (<90% of the prescribed dose) tumor progression of lymph nodes, soft tissue nearby or within the pancreas. “Distant tumor recurrence” was defined as any other tumor progression.

Overall survival (OS) was defined as time from the start of radiotherapy until death. Local control (LC) was defined from the start of radiotherapy until local tumor recurrence or last imaging available. Locoregional control (LRC) was defined from the start of radiotherapy until local or regional tumor recurrence or last imaging available. Progression free survival (PFS) was defined from the start of radiotherapy until any tumor progression or death or last imaging available.

### Toxicity

Symptoms and complaints were graded according to the International Common Terminology Criteria for Adverse Events of the National Cancer Institute (NCI CTC AE), Version 5. Toxicity rates were extracted from the charts before the start of radiotherapy, during radiotherapy and at all available time points after the end of radiotherapy.

### Statistics

OS, LC, LRC, and PFS were analyzed using the Kaplan–Meier method. Statistics and figures were performed with SPSS Statistics, version 27 (International Business Machines Corporation: IBM, New York, USA).

### Ethics

The study was approved by the Ethics Committee of the University of Heidelberg, Germany (S-688/2020).

## Results

### Patient and Treatment Characteristics

**Figure 2:**
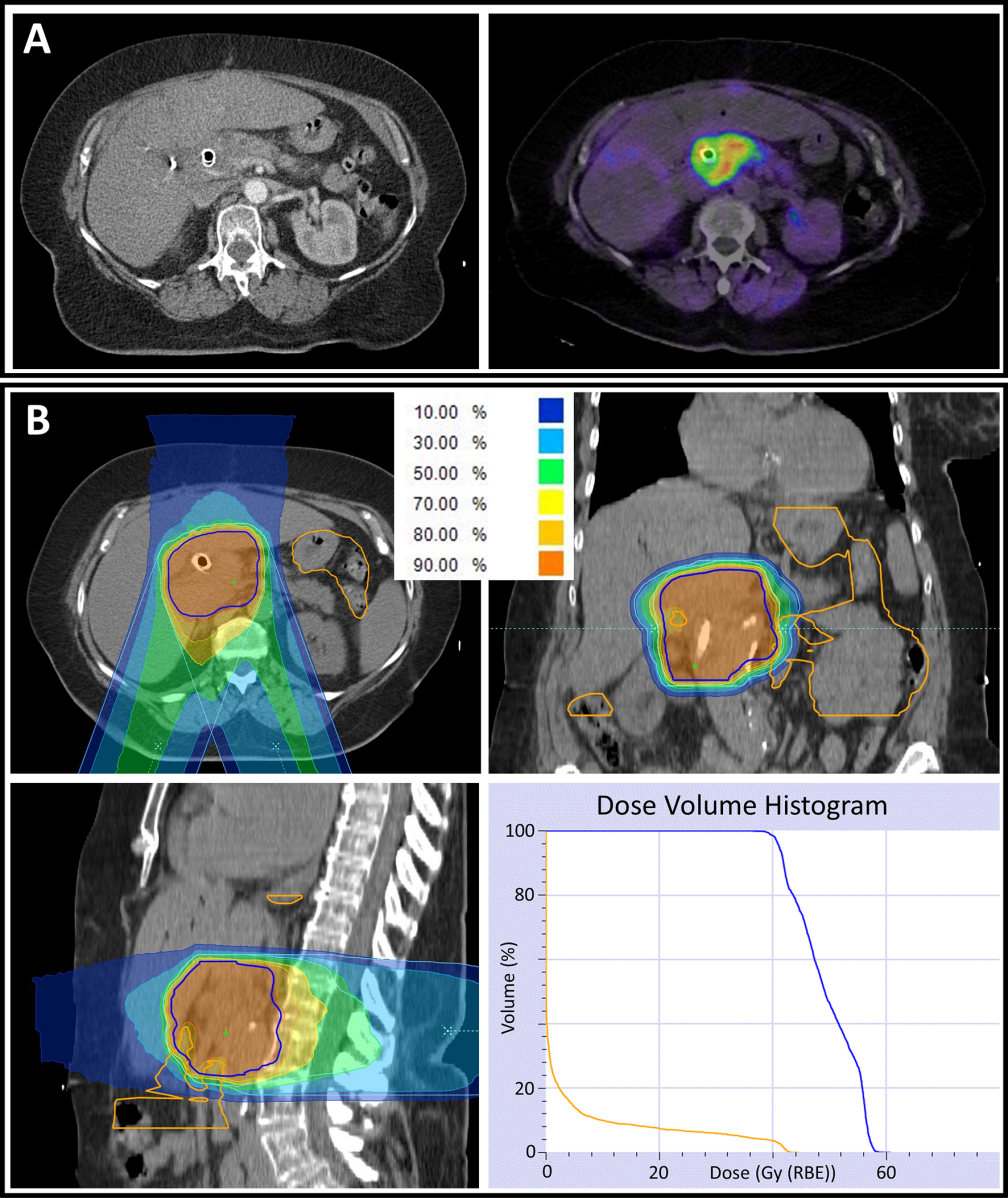
**(A)** Computed tomography (CT) scan of a locally advanced pancreatic cancer patient demonstrating a pancreatic tumor mass with an implanted biliary metal stent (left). To improve target volume definition in radiation planning, a fibroblast activation protein inhibitor-positron emission tomography (FAPI-PET) with a high tumor to background contrast was performed and matched with CT data (right). **(B)** Radiation plan of the same patient in axial (upper left), coronal (upper right) and sagittal (lower left) CT slices. The percentages of the different-colored isodose lines correspond to the prescribed dose of 48 Gy (RBE) in 12 fractions. For all volumes, an *α*/*β*-ratio of 2 Gy was used in the underlying relative biological effectiveness model. Partially, underdosage of the planning target volume (PTV, delineated in blue) needed to be accepted to respect the gastrointestinal (GI, delineated in orange) constraints. The dose volume histogram (lower right) demonstrates a ‘stereotactic-radiotherapy-like’ dose escalation within the PTV while respecting the GI constraints.

A total of 21 patients could be included in the analysis. Fourteen patients were treated with chemotherapy and one patient underwent partial tumor resection by whipple procedure prior to radiation. Prior chemotherapy regimen was heterogenous with a median of five cycles of FOLFIRINOX chemotherapy (range 1–10). Initial chemotherapy was predominantly performed as treatment of choice in LAPC patients or as neoadjuvant therapy to gain secondary operability. The majority of the patients presented in our institution due to missing tumor remission under chemotherapy or to avoid further chemotherapy. Radiotherapy with carbon ions was performed between January 2015 and July 2020. A total dose of 48 Gy (RBE) was delivered in 12 fractions in all cases. In three cases, concomitant chemotherapy with gemcitabine 300 mg/m^2^ body surface was administered weekly (2–3 cycles). Patients that were known non-responder to gemcitabine and patients that could not receive chemotherapy due to their general condition were not treated with concomitant chemotherapy. Additionally, the combination of gemcitabine chemotherapy and carbon ion radiotherapy was not tested to be safely applicable in 2015 which resulted in restrictive concomitant chemotherapy prescription at our institution. After radiotherapy, two initially inoperable patients were operated. One patient underwent total pancreatectomy (R1) after having developed local tumor recurrence after radiotherapy. In the second case, the tumor was deemed unresectable during operation and the procedure was completed without resection. During both operations, slight fibrosis was seen without leading to any interventional complications. Accordingly, the overall secondary resection rate was 5%. In case of tumor progression during follow-up, patients were treated with different chemotherapy and immunotherapy regimen. Four patients were treated with gemcitabine and nab-paclitaxel, two patients with FOLFIRINOX, and further two patients with a combination of ipilimumab, nivolumab, and maraviroc. Detailed patient and treatment characteristics are summarized in **Tables 1** and **2**. A representative radiation plan is shown in **Figure 2**.

**Table 1:** Patient characteristics.

	n	(%)
**Number of patients**	**21**	**(100)**
Sex		
Male	16	(76)
Female	5	(24)
Age at radiotherapy (median in years, range)	70 (48–83)	
Localization of initial pancreatic cancer		
Pancreatic head	13	(62)
Pancreatic body	7	(33)
Pancreatic tail	1	(5)
Initial AJCC* stage		
IIB	1	(5)
III	17	(81)
IV	3	(14)
Prior chemotherapy		
FOLFIRINOX°	10	(48)
FOLFIRINOX°, followed by gemcitabine + nab-paclitaxel	4	(19)
None	7	(33)
Time in months: prior chemotherapy (median, range)	5 (1–10)	
Prior surgery		
Whipple procedure (R2 resection)	1	(5)
None (apart from biopsy)	20	(95)
Histology		
Ductal adenocarcinoma	18	(86)
unknown	3	(14)
Secondary resection	1	(5)

*AJCC, American Joint Committee on Cancer.

°FOLFIRINOX, chemotherapy regimen consisting of folinic acid, fluorouracil, irinotecan, and oxaliplatin.

**Table 2:** Treatment characteristics.

	n	(%)
**Radiotherapy**	**21**	**(100)**
Time in months: diagnosis to radiotherapy (median, range)	8 (2–13)	
Pre-radiotherapy AJCC* stage		
III	19	(91)
IV	2	(9)
Radiation technique		
Carbon ions, active raster-scanning	21	(100)
Prescribed dose		
48 Gy (RBE) in 12 fractions	21	(100)
Concurrent chemotherapy		
Gemcitabine 300 mg/m^2^ body surface weekly	3	(14)
None	18	(86)
Patient position		
Supine	15	(71)
Prone	6	(29)
Volume in ccm (median, range)		
GTV (Gross tumor volume)	43.6 (13.0–129.7)	
CTV (Clinical target volume)	128.4 (26.1–323.3)	
ITV (Internal target volume)	183.4 (48.3–583.5)	
PTV (Planning target volume)	303.2 (96.7–812.0)	
Number of radiation beams		
2	21	(100)
*α*/*β*-ratio used in local effect model (LEM) I		
2 Gy	3	(14)
5 Gy	18	(86)

*AJCC, American Joint Committee on Cancer.

### Survival and Tumor Control

**Figure 3:**
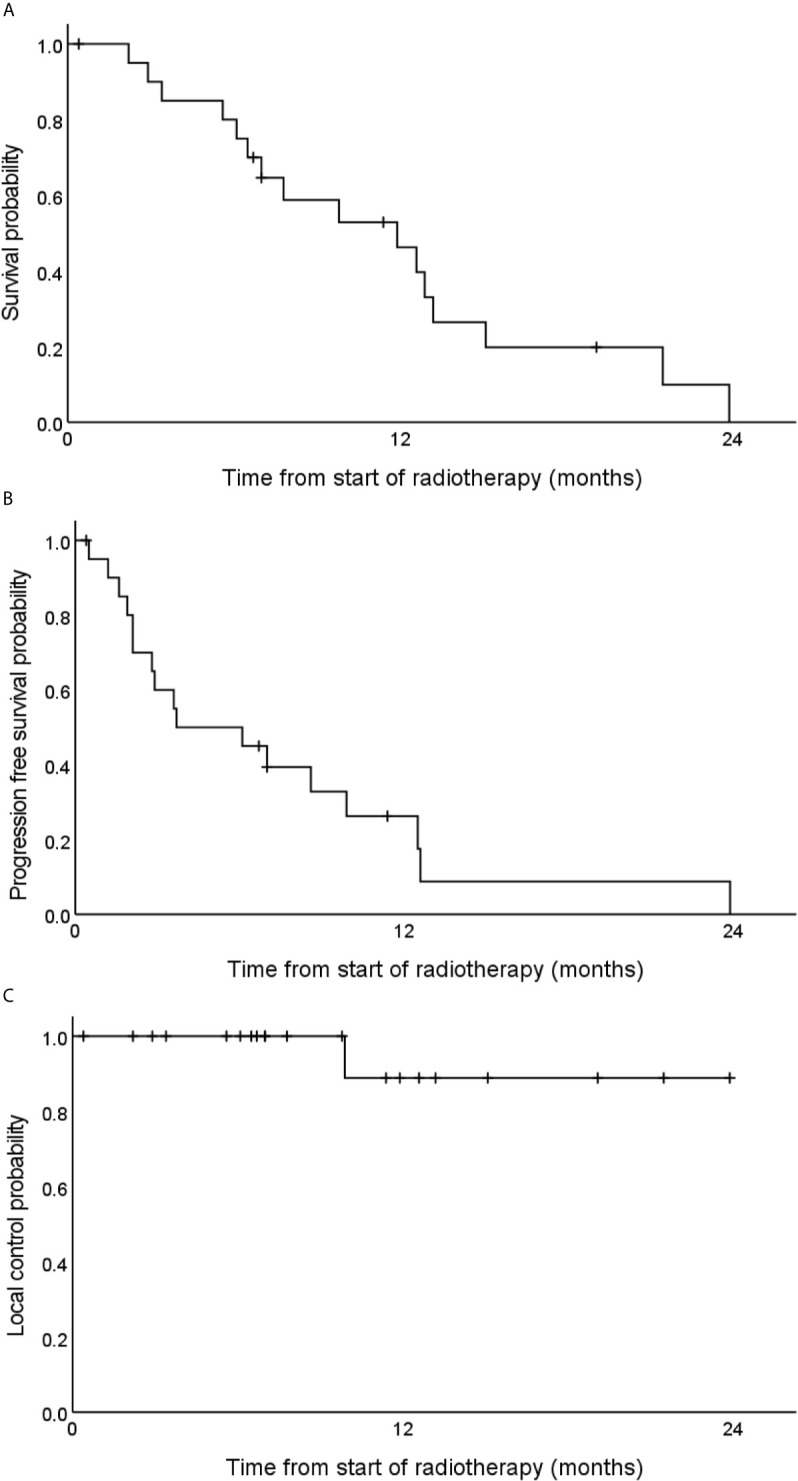
Estimated overall survival **(A)**, progression free survival **(B)** and local tumor control **(C)** rates of 21 locally advanced pancreatic cancer patients after carbon ion radiotherapy with 48 Gy (RBE) in 12 fractions.

After a median follow-up time of 19.1 months, the estimated 1-year OS rate was 40.0% (**Figure 3A**). Two years after the start of radiotherapy, two of the observed patients were still alive and three patients were lost to follow-up. The observed median overall survival was 11.9 months. The corresponding 95% confidence interval (CI) was 6.0–17.8 months. The estimated 1-year PFS rate was 10% (**Figure 3B**), and the median PFS was 3.7 months (95% CI 0.0–9.2).

Local progression could be observed in one patient 10 months after the start of radiotherapy (**Figure 3C**). The corresponding tumor could be resected but two months later distant metastases occurred. Regional and distant tumor recurrences were simultaneously observed in another patient that was treated with chemotherapy thereafter. No further locoregional tumor recurrence could be observed. One-year local control and one-year locoregional control rates were 89 and 84%, respectively.

### Toxicity

During radiotherapy, there was an increase of reported nausea (13% before radiotherapy *vs*. 48% during radiotherapy) and diarrhea. Nevertheless, these symptoms decreased after radiotherapy. One patient developed ascites CTCAE grade III during radiotherapy. Histopathological examination revealed underlying metachronous peritoneal carcinomatosis. No further higher-graded toxicity could be observed. After radiotherapy, 14% of the patients reported on fatigue. Toxicity rates are shown in **Table 3**.

**Table 3:** Toxicity rates.

Symptoms (NCI CTCAE grades)	Before RT* n (%)	During RT* n (%)	After RT* n (%)
Abdominal pain			
I	5 (24)	5 (24)	3 (14)
II	5 (24)	4 (19)	2 (9)
Gastric hemorrhage			
II	0	0	1 (5)
Diarrhea			
I	2 (9)	3 (14)	1 (8)
Ascites			
II	0	0	1 (5)
III	0	1 (5)	1 (5)
Nausea			
I	2 (9)	6 (29)	1 (5)
II	1 (5)	4 (19)	3 (14)
Dermatitis			
I	0	2 (9)	0
Fatigue			
I	0	2 (9)	1 (5)
II	0	0	2 (9)
No complaints	10 (48)	7 (33)	6 (29)

*RT, radiotherapy.

## Discussion

To the best of our knowledge, we present the first European data on carbon ion radiotherapy in LAPC. The observed results demonstrate that carbon ion radiotherapy in pancreatic cancer is both feasible and well tolerable. Whereas convincing local tumor control rates could be achieved, OS rates were low due to a very short PFS of 3.7 months since distant metastases occurred early in most patients.

In all cases, radiation was completed as intended, and no radiation-induced higher graded toxicity was seen. Observed nausea and diarrhea could adequately be explained by radiation-induced mucositis of the gastrointestinal tract. Accordingly, these two symptoms decreased after the end of radiotherapy. Those findings are in line with the current literature. There are two retrospective analyses regarding carbon ion radiotherapy in LAPC (24, 25). Furthermore, Shinoto et al. published results of a prospective phase II dose-escalating trial of 76 patients (15). In the mentioned studies, the most common toxicities included anorexia and gastrointestinal ulcer or bleeding. The latter could be observed in <3% of the patients. In the present analysis, only one grade II bleeding of the lower gastrointestinal could be observed during follow-up. The patient was treated with anticoagulant therapy at the time of the event so that a correlation with the performed radiation seems to be less probable.

In the only prospective trial published so far, Shinoto et al. demonstrated a 1- and 2-year local tumor control rate of 92 and 83%, based on CT imaging (15). We could confirm the local effectiveness of carbon ion radiotherapy with an observed 1-year local control rate of 87%.

Nevertheless, OS results differed considerably. Shinoto et al. observed a median OS of 19.6 months (15). Kawashiro et al. presented an even higher median OS of 21.5 months after carbon ion radiotherapy in LAPC patients (25). This is almost twice as high as the observed 11.9 months of the present study. In the LAP 07 trial which compared photon radiotherapy-based chemoradiation with chemotherapy after induction therapy, the observed median overall was 12.8 months (4). Compared to these historical findings, we did not observe an OS improvement by carbon ion radiotherapy over photon radiotherapy in the present study.

One difference between the Japanese data and the present analysis is the underlying radiation dose concept. Kawashiro et al. irradiated with total doses of up to 55.2 Gy (RBE) delivered in 12 fractions. Furthermore, they described a correlation of higher-dosed radiotherapy and OS improvement (25). In the present analysis, we irradiated with a total dose of 48 Gy (RBE) in 12 fractions. However, dose concepts of different carbon ion facilities cannot be compared directly, which aggravates the interpretation of these findings. To be able to compare the approach at the Heavy-Ion Medical Accelerator (HIMAC) at the National Institute of Radiological Science (NIRS) in Japan with the LEM I-based approach at our institution, Steinstrater et al. published conversion tables (20). According to these assumptions, the irradiated maximum single doses of 4.6 Gy (RBE) by Kawashiro et al. should correlate with single doses of 4.4–5.0 Gy (RBE) at our institution. Altogether, the radiation dose concept at our institution [single dose: 4.0 Gy (RBE)] is supposed to be lower than the maximum one used in Japan. Nevertheless, considering the comparable local tumor progression rates of the different analyses, it seems rather unlikely that the diverse radiation dose concepts satisfactorily explain the OS differences.

Shinoto et al. and Kawashiro et al. performed elective nodal irradiation and included the neuro-plexus region in the CTV (15, 25). At our institution, carbon ion radiotherapy volumes were kept as small as possible without elective nodal irradiation. The latter could lead to a higher rate of tumor recurrences. However, we did not observe high rates of locoregional tumor progression which partially contradicts this hypothesis.

Another significant difference between the Japanese trials and our study is the administration of chemotherapy. Shinoto et al. and Kawashiro et al. combined carbon ion radiotherapy with gemcitabine-based chemotherapy using doses of up to 1,000 mg/m^2^. In the present study, only 14% of the patients were treated with simultaneous chemotherapy and administered gemcitabine doses were lower (300 mg/m^2^). The missing chemotherapy in the present treatment concept could explain the observed high rate of distant tumor progression and the relatively low OS rate.

Finally, there is a selection bias in the presented patient cohort. Since the final publication of the LAP 07 trial results, LAPC patients in Europe are predominantly treated with chemotherapy. Patients that are capable of being treated with intense chemotherapy regimen such as FOLFIRINOX will typically not be assigned to radiotherapy unless they did not respond to chemotherapy pretreatment. Sixty-seven percent of the irradiated patients of the present analysis were pre-treated with intense chemotherapy regimen and did not or did only poorly respond to pre-treatment. The median time from initial diagnosis to irradiation was 8 months. Altogether, the irradiated patient cohort consisted mostly of non-responding patients to pre-treatment with chemotherapy.

This selection bias could possibly explain the observed limited OS of the present study. The low secondary resection rate of 5% supports this hypothesis. In a large meta-analysis of LAPC patients being treated with different modalities, the secondary resection rate was approximately 25% (8). Furthermore, the median gross tumor size of 43.6 ccm in the present analysis is almost three times larger than the observed tumor size of the prospective trial of Shinoto et al. (14.8 ccm) (15) indicating a negative selection bias of the present patient cohort, too.

The present study has several limitations. First, the quality of data acquisition is limited because of the retrospective character of the analysis. Several patients did not regularly perform follow-up examinations, and no standardized quality of life questionnaires were used. Second, a sample size of 21 patients is small, which is due to the limited number of carbon ion facilities making carbon ion radiotherapy a rare treatment option. Third, the observed high local tumor control rate could be biased by the high rate of distant tumor progression during follow-up examination. It is possible that patients did not reach the criteria of local tumor progression because they deceased early after the end of radiotherapy.

On the other hand, a strength of the analysis is the reliability of the estimated overall survival due to the high number of reported deaths. Furthermore, the observed local tumor control rate and the toxicity rates seem to confirm the radiation dose concept as only one patient developed local tumor progression and no higher-graded radiation-induced toxicity was seen.

In conclusion, carbon ion radiotherapy in pancreatic cancer is well tolerable and locally effective. In the present analysis, an expected OS benefit over historical photon radiotherapy data could not be observed. This seems to be due to a negative selection bias of the described patient cohort. Considering the high rate of distant tumor progression, carbon ion radiotherapy should be combined with chemotherapy in future studies.

## Data Availability Statement

The raw data supporting the conclusions of this article will be made available by the authors, without undue reservation.

## Ethics Statement

The study was approved by the Ethics Committee of the University of Heidelberg, Germany (S-688/2020). Written informed consent for participation was not required for this study in accordance with the national legislation and the institutional requirements.

## Author Contributions

JL and KH designed and directed the project. JL gathered the data. JL analyzed the data and wrote the manuscript. PN, FW, PH, JD, and KH helped in finalizing the manuscript. JD and KH supervised the project. All authors contributed to the article and approved the submitted version.

## Funding

JL is funded by the Physician-Scientist Program of Heidelberg University, Faculty of Medicine. We acknowledge financial support by the Open Access Publishing Fund of Heidelberg University.

## Conflict of Interest

JD received grants from Viewray Incorporated, The Clinical Research Institute GmbH (CRI), Accuray International Sarl, RaySearch Laboratories AB, Vision RT Limited, Merck Seono GmbH, Astellas Pharma GmbH, Astra Zeneca GmbH, Siemens Healthcare GmbH, Solution Akademie GmbH, Egomed PLC Surrey Research Park, Quintiles GmbH, Pharmaceutical Research Associates GmbH, Boehringer Ingelheim Pharma GmbH&CoKG, PTW-Freiburg Dr. Pychlau GmbH, Nanobiotix SA, Accuray Incorporated, Bristol-Myer Squibb GmbH&CoKG aA and Merck KHG aA. As chairman of HIRO (Heidelberg Institute of Radiation Oncology, Heidelberg, Germany) and a managing director of the NCT (National Center for Tumor Diseases) Heidelberg, Germany, Juergen Debus is responsible for collaborations with a multitude of companies and institutions. Juergen Debus is CEO of the HIT Betriebs-GmbH and a member of the board of trustees of the Physikalisch-Technische Bundesanstalt (PTB). He attended advisory board meetings of MERCK KGaA (Darmstadt).

The remaining authors declare that the research was conducted in the absence of any commercial or financial relationships that could be construed as a potential conflict of interest.
